# Electrohydrodynamic redox printing vs. physical vapour deposition: a comparative study of nanoporous Ag morphology and SERS performance

**DOI:** 10.1186/s11671-025-04409-1

**Published:** 2025-12-09

**Authors:** Nikolaus Porenta, Loredana Piazza, Ralph Spolenak

**Affiliations:** Department of Materials, Laboratory for Nanometallurgy, ETH Zürich, Vladimir-Prelog-Weg 1-5/10, 8093 Zürich, Switzerland

**Keywords:** Nanoporous metal, Surface enhanced Raman spectroscopy, Additive manufacturing, FIB tomography, Thin film

## Abstract

The increasing demand for miniaturised, high-performance sensing platforms necessitates materials that can be deposited with high spatial precision. Surface-enhanced Raman spectroscopy (SERS) has emerged as a powerful analytical technique, offering significant signal amplification. Nanoporous (np) metals, particularly np Ag, are promising candidates for SERS substrates due to their high surface area and tunable nanostructure. In this study, we compare np Ag fabricated via electrohydrodynamic redox printing (EHD-RP), an additive manufacturing technique with high spatial resolution, to conventionally produced counterparts using physical vapour deposition (PVD). EHD-RP-derived np Ag exhibits comparable SERS performance to PVD samples. Structural analysis reveals that the density of sub-25 nm pores and the degree of structural disorder strongly contribute to enhancement factors. Additionally, EHD-RP-derived np Ag demonstrates excellent stability under varying illumination conditions and effectively catalyses the plasmon-driven dimerisation of 4-nitrobenzenethiol. These results underscore the potential of EHD-RP for fabricating functional nanostructured materials for integrated sensing applications.

## Introduction

Since its discovery in 1928 [1, 2], Raman spectroscopy has been developed into a powerful and versatile analytical tool, with applications spanning a wide range of scientific disciplines. These include, among others, the identification and preservation of cultural heritage materials [3], forensic investigations [4], biomedical diagnostics [5], and mapping of strain distributions in materials [6]. However, despite its utility, Raman spectroscopy has long been limited by inherently low scattering cross-sections, resulting in weak signal intensities that hinder sensitivity [7].

This limitation was overcome with the discovery of surface-enhanced Raman spectroscopy (SERS) by Fleischmann et al. in 1974 [8]. By employing roughened Ag electrodes as substrates, they observed a significant increase in Raman signal intensity. Subsequent research identified two principal mechanisms responsible for this enhancement: electromagnetic (EM) enhancement and charge transfer (CT) [9–11]. The EM mechanism is enabled by the excitation of localised surface plasmon resonances (LSPRs) in metallic nanostructures, which generate regions of intense electromagnetic fields, so-called hot spots that amplify the Raman signal of molecules located nearby. The CT mechanism arises from electronic interactions between the metal substrate and the adsorbed analyte, specifically involving electron transfer to the lowest unoccupied molecular orbital of the molecule. These combined mechanisms allow for highly sensitive detection, with SERS enabling even single-molecule detection with optimised substrate geometries [12, 13].

Among the various classes of nanostructured SERS substrates, nanoporous (np) metals have emerged as particularly promising candidates, due to their ease of fabrication [14, 15]. Their bicontinuous network of nanoscale ligaments and pores yields a large surface area and a high density of potential hot spots. The morphology of the np structure has a direct influence on SERS performance. For instance, smaller pore sizes, down to a few nanometres, have been correlated with higher enhancement factors (EF), attributed to stronger field confinement and more numerous hot spots [16–19]. Additionally, recent studies suggest that structural disorder within the network can modulate the local electromagnetic field distribution, potentially increasing the number of hot spots and improving overall enhancement [20–22].

In this study, we investigate how different fabrication methods can influence the SERS performance of np Ag. For this two methods with different deposition mechanics were chosen: physical vapour deposition (PVD) and electrohydrodynamic redox 3D printing (EHD-RP). PVD is a well-established method in both research and industry [23], wherein metal atoms in the vapour phase condense onto a substrate, forming a thin film as the adatoms diffuse to energetically favourable positions. In contrast, EHD-RP is a recently developed additive manufacturing technique for metals. This technique allows for nanoscale voxel-by-voxel deposition [24]. In this method, a quartz nozzle with an orifice diameter of approximately 100 nm, filled with an aqueous mixed metal salt solution, is positioned near a substrate. Upon application of an electric field, metal-ion-loaded droplets are ejected from the nozzle and deposited onto the substrate, where the ions are reduced and the solvent evaporates. EHD-RP offers several advantages, including high geometric design freedom [25], compatibility with a variety of substrates [26], and precise compositional control through control of the salt composition in solution [27].

Here, we compare the SERS EFs of np Ag networks fabricated by PVD and EHD-RP as a function of the initial alloy composition and benchmark these results against roughened Ag thin films. To understand the origin of observed differences in SERS activity, the structural characteristics of the resulting np networks are analysed in detail using focused ion beam (FIB) tomography. Furthermore, we demonstrate the applicability of EHD-RP-derived np Ag as an active catalyst in plasmon-driven chemical reactions, highlighting its potential as a multifunctional material for sensing and catalysis applications.

## Materials and methods

### Sample fabrication

#### Substrate preparation

The procedure for substrate preparation has been described in detail in previous literature [27, 28]. In short, 80 nm of Au (MaTeck, 99.99%, 7.62 cm diameter) were deposited on $\textrm{Si}_3\textrm{N}_4$ coated Si wafers (SiMat) with a 5 nm Ti (MaTeck, 99.995%, 7.62 cm diameter) adhesion layer in-between. The finished wafers were then cut into smaller pieces before being used as substrates.

#### Printing procedure

Mixed aqueous solutions of $\textrm{CuSO}_4$ and $\textrm{Ag}_2\textrm{SO}_4$ were used for printing. These solutions were prepared from a 4 mM $\textrm{CuSO}_4$ . 5 $\textrm{H}_2O$ (Sigma Aldrich, 99.999% metal basis) and a 2 mM $\textrm{Ag}_2\textrm{SO}_4$ (Alfa Aesar, 99.999% metal basis) stock solution. The solutions were mixed with high-purity water (Fisher Chemical, Optima^®^) to achieve a total metal ion concentration of 1 mM. Compositions of Cu:Ag 6:4, 7:3, 8:2 and 9:1 were fabricated.

The solutions were filled into quartz nozzles with opening sizes of approximately 80 nm. These nozzles were fabricated in-house. A Au wire was submerged into the solution and connected to a power supply (Keysight, B2962). The substrate was then also connected to the power supply. All prints were conducted at a constant current of 1 nA, the applied potential was varied to achieve the constant current. A more detailed description of the printing setup used in this work can be found in previous literature [27, 28].

#### PVD sputtering procedure

Co-sputtering was used to deposit CuAg films using PVD DC magnetron sputtering (PVD Products Inc.) from a Cu (MaTeck, 99.99%, 7.62 cm diameter) and a Ag target (MaTeck, 99.99%, 7.62 cm diameter). All depositions were conducted at a base pressure below $5 \cdot 10^{-7}$ Torr, with a deposition pressure of 3 mTorr and 30 rpm substrate rotation. The applied power for Cu was set to 271 W and the Ag power was varied to achieve different compositions. The Ag power used was 73 W, 43 W and 19 W to achieve Cu:Ag ratios of 7:3, 8:2 and 9:1, respectively. These powers were calculated using the same procedure as described in previous literature [28, 29].

Pure Ag films were deposited at the same deposition parameters, except for using an applied power of 150 W.

#### Dealloying procedure

All CuAg alloy samples were dealloyed electrochemically in 10 vol% $\textrm{H}_3\textrm{PO}_4$ solutions. These solutions were prepared from concentrated $\textrm{H}_3\textrm{PO}_4$ (Sigma Aldrich) and high purity water. An Autolab PGSTAT12 FRA2 potentiostat (Metrohm) was used to apply $-0.15$ V against a saturated mercury sulphate electrode (SMSE, Pine Research, RREF0025) as a reference electrode with a glassy carbon counter electrode. This potential was chosen according to previous literature [24]. To verify the literature value a circular voltammetry (CV) measurement of a mixed 8 mM $\textrm{CuSO}_4$ and 1 mM $\textrm{Ag}_2\textrm{SO}_4$ solution in 10 vol% $\textrm{H}_3\textrm{PO}_4$ was conducted. The acquired CV curve is shown in Fig. S1.

#### Ag roughening

Deposited Ag films were etched in 4.8 M $\textrm{HNO}_3$ for 10, 30 and 37 s. The etching solution was prepared from concentrated $\textrm{HNO}_3$ (Alfa Aesar, 99.9999% metal basis) and high purity water.

### Analysis

#### Electron microscopy

All scanning electron microscopy (SEM) analyses were conducted on a Magellan 400 (Thermo Fisher Scientific). An acceleration voltage of 5 kV and a beam current of 50 pA were used.

#### Focused ion beam tomography

FIB tomographies were obtained on a Helios 600i (Thermo Fisher Scientific) using the Auto Slice & View 4 Software (AS&V4). As a preparatory step, the sample was infiltrated with carbon using an electron beam assisted deposition with 30 kV acceleration voltage and 1.4 nA beam current. A region of interest (ROI) of 4.5 $\mu$m width was chosen. The ROI was then covered with a 1 $\mu$m thick carbon protective layer using ion beam assisted deposition with 30 kV acceleration voltage and 0.8 nA beam current. A cross section of 2 $\mu$m depth was cut below the carbon covered region with 30 kV acceleration voltage and 0.8 nA beam current. Following this, a cleaning cross section was done with 30 kV acceleration voltage and 40 pA beam current. Vertical drift was automatically corrected for, using the "y-shift correction" feature in AS&V4. Tomography images were acquired using the electron beam with 2 kV acceleration voltage and 0.17 nA beam current in the through-lens-detector mode. The system was tilted to $52^\circ$ with tilt correction and dynamic focus enabled. Ion beam milling was performed at 30 kV acceleration voltage and 40 pA beam current, leading to a slice thickness of approximately 7.5 nm.

#### Atomic force microscopy

Surface roughness of PVD sputtered Ag films was measured with atomic force microscopy (AFM), using an Asylum Research Cypher scanning probe microscope operated in contact mode. Samples were mounted on magnetic holders with silver paste and placed inside the AFM system, which was equipped with Arrow-UHF-10 tips (Nanoworld). For each sample, set-point and gain parameters were adjusted individually, to ensure optimal measurement conditions. All scans were done over an area of 2 $\mu$m $\times$ 2 $\mu$m. The Gwyddion SPM data analysis software (Version 2.67) was used to calculate the root mean square (RMS) roughness and the surface area. For each sample, calculations were performed on three distinct areas, and the mean of these values was subsequently used for further analysis.

The software was also used for data visualisation.

#### Raman spectroscopy

Two probe molecules, 4-nitrobenzenethiol (4-NBT, Fluorochem) and 4-mercaptobenzoic acid (4-MBA, Fluorochem), were used for SERS measurements. Samples were immersed in 0.1 mM solution for 4-NBT and 10 mM for 4-MBA for 2 h to form a self-assembled monolayer (SAM) before SERS measurements. The samples were rinsed with technical ethanol to remove excess molecules upon removal from the solution.

SERS experiments were performed on a WiTec alpha300 R system with a 100x objective (NA $=$ 0.8) and at laser wavelength of 532 nm. Assuming an Airy-disk-like laser spot the spot size is given as $1.22\frac{\lambda }{NA}$, resulting in a spot size of approximately 811 nm. A laser power of 200 $\mu$W, integration time of 8 s, and one accumulation were used to measure the SERS signal. For the laser power studies the integration was varied to not saturate the detector. To account for this all signal intensities were normalised by the integration time. As a reference pure 4-MBA powder was measured on the same Raman system with the same parameters.

To calculate the EF the following equation was used:

1 $$\begin{aligned} EF=\frac{I_{SERS}}{I_{Raman}}\times \frac{N_{Raman}}{N_{SERS}} \end{aligned}$$

where $I_{SERS}$ and $I_{Raman}$ are the measured signal intensities of a Raman band in SERS and Raman measurements, respectively. In this work we used the symmetric C=C vibrational mode at approximately 1587 cm^-1^ (for further peak assignment see section 3) for EF calculations. In the calculation an averaged intensity value of at least three SERS measurements was used as $I_{SERS}$. $N_{Raman}$ and $N_{SERS}$ are the number of probe molecules in the beam spot for the Raman and SERS measurements, respectively. $N_{Raman}$ was calculated from the density of 4-MBA powder, assuming a spherical laser spot. To calculate $N_{SERS}$ the surface area in the laser volume needs to be known. For np Ag the surface area per unit volume was calculated from FIB tomographies and used to estimate the surface area within the laser-irradiated volume, assuming a spherical laser spot geometry. For roughened Ag samples surface area values were taken from AFM measurements. Furthermore, the SAM surface coverage needs to be known. For 4-MBA a surface coverage of approximately 0.5 nmol cm^-2^ has been reported on flat Au substrates [30, 31]. Np metal substrates typically exhibit reduced SAM packing densities. Literature reports indicate that surface coverages on such structures are approximately 60% of those observed on planar surfaces [32–34]. Accordingly, this correction factor was applied to adjust the estimated surface coverage on np Ag samples.

#### Data processing

Tomography reconstruction was done using Fiji version 2.14.0/1.54f and the BoneJ plugin version 7.0.19. Image sequences were horizontally aligned through registration using Stackreg and vertically aligned using a custom Python script. The aligned stack was cut to a ROI, and background subtraction was applied using a rolling ball radius of 50 px. Then, the stack was blurred using a Gaussian blur ($\sigma$ $=$ 1), and the Otsu algorithm was applied to binarize the stack for further processing. The morphological operation "Open" and the "Purify" feature of BoneJ were used to remove unwanted noise and non-connected ligaments. For all samples, the ROI was set in the middle of the printed pads and was constant in size (576 $\times$ 56 px). Ligament and pore sizes were determined using the "Thickness" feature to calculate the mean, standard deviation, and maximal values of ligament and pore sizes. Ligament and pore size distribution were obtained by analyzing the histogram of the thickness maps generated by BoneJ. Pores larger than 200 nm were excluded from the analysis due to the onset of surface cracks at this value. The full width at half maximum (FWHM) of the distribution was determined by fitting a Weibull distribution to the data and calculating the FWHM from the fit. Further data obtained from BoneJ includes, surface area, ligament volume, and relative density.

Further data analyses and plotting were done using RStudio: Integrated Development Environment for R (Version 2024.04.0+735).

## Results and discussion

### Sample morphologies

**Fig. 1:**
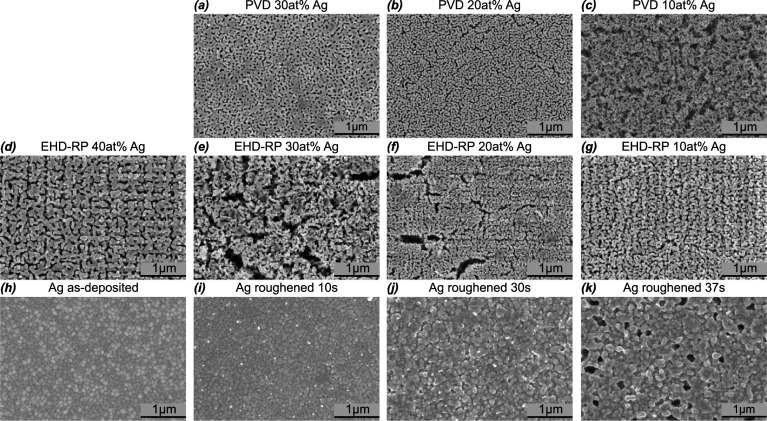
Surface morphologies of np Ag and roughened Ag films.** a**–**c** SEM micrographs of np Ag fabricated from PVD samples with Ag starting content of 30, 20 and 10 at%, respectively.** d**–**g** SEM micrographs of np Ag fabricated from EHD-RP samples with Ag starting content of 40, 30, 20 and 10 at%, respectively.** h**–**k** SEM micrographs of Ag films as-deposited and roughened for 10, 30 and 37 s, respectively

Figure 1 presents representative SEM micrographs of np Ag and roughened Ag samples investigated in this study. The composition of the used starting alloys were confirmed by SEM EDX measurements. The measured compositions are shown in Tab. S1. For the PVD-derived samples, a clear dependence of the resulting microstructure on the initial Ag content is observed. Specifically, a reduction in the initial Ag content results in a decreased amount of residual dense material following dealloying. At 30 at% Ag, substantial regions of dense material remain visible. These regions are no longer apparent in the 20 at% Ag sample, which exhibits a homogeneous bicontinuous network composed of similarly sized ligaments and pores. In contrast, the 10 at% Ag sample displays pronounced cracking and a more disordered porous network. The EHD-RP samples exhibit a less straightforward trend with respect to initial Ag content. At 40 at% Ag, a crack-free np Ag structure is observed, characterised by both vertical and horizontal alignment of ligaments. Upon decreasing the Ag content to 30 at%, cracking begins to appear, accompanied by a reduction in structural density and order. Additionally, small, darker regions, presumed to be impurities, become apparent. Further reduction to 20 at% Ag leads to the formation of a denser, but still cracked, np network. At 10 at% Ag, the EHD-RP sample again forms a dense, crack-free network exhibiting a well-defined, ordered morphology reminiscent of the 40 at% sample. The as-deposited pure Ag film exhibits a dense microstructure. Upon roughening for 10 s, the emergence of small protrusions – visible as bright spots in the SEM micrographs – indicates the onset of surface modification. The roughness of the surface increases with roughening time, as evidenced by increasingly pronounced grain boundaries. After 30 s of treatment, the surface becomes notably uneven, and small holes begin to form. These features grow further with extended roughening up to 37 s, leading to a higher density of holes. To quantify surface roughness, AFM measurements were performed; corresponding micrographs are shown in Fig. S2. The RMS surface roughness increased monotonically with roughening time, from 4.1 ± 0.3 nm for the as-deposited sample to 4.3 ± 0.1 nm, 11.6 ± 0.9 nm, and 16.7 ± 0.1 nm for 10 s, 30 s, and 37 s of roughening, respectively.

No 40 at% Ag PVD sample could be fabricated, as the applied dealloying conditions were insufficient to induce dealloying. It has been reported that an increased concentration of the more noble metal elevates the critical potential required for dealloying [35]. Below this critical potential, the noble metal tends to form a passivating surface layer that inhibits further dealloying [36]. Evidence of this passivation behaviour is also observed in the 30 at% Ag PVD sample, as shown by the dealloying current measurement in Fig. S3. The current initially rises and then gradually decreases; however, this decline does not continue until no more current can be measured. Instead, the current subsequently increases, indicating the continuation of the dealloying process. The initial decrease may be attributed to the formation of a passivating layer, which temporarily retards etching until a bicontinuous network is established, thereby enabling the continuation of dealloying. The resulting morphologies of the PVD-derived np networks exhibit the expected trend: as the Ag content in the precursor alloy decreases, the amount of remaining material in the final np structure decreases correspondingly. EHD-RP samples dealloyed substantially faster than their PVD counterparts. As evidenced by the dealloying currents shown in Fig. S3, dealloying occurs in under 1 s. This disparity arises primarily from the different dimensions of the as-deposited films. While both sample types had an approximate thickness of 800 nm, the PVD samples measured 0.5 cm $\times$ 0.4 cm in lateral dimensions, whereas the EHD-RP samples comprised only three to four 10 $\mu$m $\times$ 10 $\mu$m pads. This corresponds to a volume reduction by a factor of approximately 5$\times$10^4^, thereby significantly shortening the dealloying time for EHD-RP samples. Unlike the PVD samples, EHD-RP-derived np structures do not exhibit a clear morphological trend with varying Ag content. Both the 40 and 10 at% Ag samples yielded crack-free networks with partially ordered ligament arrangements. Similar ordering has been reported in previous work on EHD-RP-fabricated np Ag, and is likely a consequence of the layer-by-layer deposition mechanism intrinsic to the EHD-RP process [28]. Conversely, the cracks observed in the 30 and 20 at% Ag EHD-RP samples are likely attributable to pre-existing defects in the as-deposited films. Such defects may act as stress concentrators and propagate during dealloying as a means of relieving internal stress [37]. For the pure Ag samples, the observed increase in surface roughness with prolonged roughening time follows the expected trend. The preferential etching along grain boundaries is consistent with literature, where etching preferentially occurs along defects [38, 39]. The formation of holes during the roughening process has also been reported in earlier studies [40, 41], although their origins were not explicitly discussed. One plausible explanation may be the orientation-dependent etching behaviour of polycrystalline Ag films. These films typically exhibit a strong (111) texture, with minor fractions of other crystallographic orientations [42, 43], which may be more susceptible to chemical attack.

### SERS properties

**Fig. 2:**
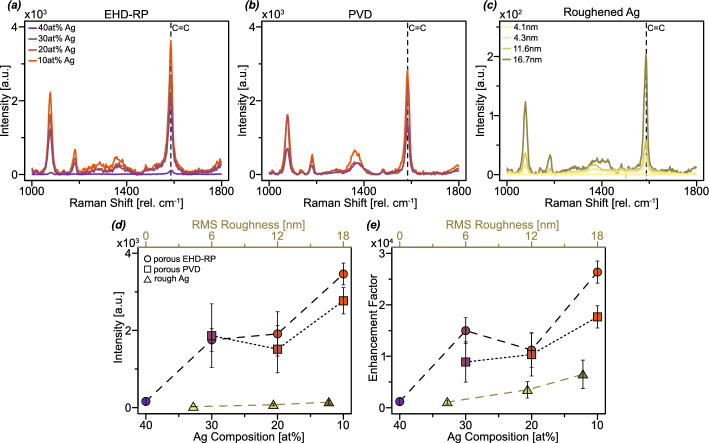
SERS signal intensities and EFs in dependence on composition and surface roughness. (**a**) SERS spectra of 4-MBA on np Ag fabricated using EHD-RP with varying compositions of the starting alloy. **b** SERS spectra of 4-MBA on np Ag fabricated using PVD with varying compositions of the starting alloy. **c** SERS spectra of 4-MBA on roughened Ag films with varying surface roughness values. **d** SERS intensities of the symmetric C=C vibration peak at 1587 cm^-1^ for different samples. Error bars correspond to the standard deviation. **e** Calculated EFs using the symmetric C=C vibration peak at 1587 cm^-1^. Error bars correspond to the error calculated from the standard deviation of intensity values using error propagation. **d, e** Round data points are EHD-RP samples, rectangular data points are PVD samples and triangular data points are roughened Ag films. For EHD-RP and PVD samples the SERS intensity and EF are plotted against the composition on the lower axis. The composition is also illustrated in the colour of data points where purple corresponds to a higher Ag content and orange to a lower Ag content. For roughened Ag films the SERS intensity and EF are plotted against the RMS roughness on the upper axis. The darker greens correspond to higher roughness values

Figure 2a–c presents SERS spectra of 4-MBA acquired on np Ag as well as on roughened Ag films. For both EHD-RP and PVD-derived np Ag samples, a clear trend of increasing signal intensity with decreasing initial Ag content in the alloy is observed. A similar dependence is seen in the roughened Ag films, where increased surface roughness leads to enhanced SERS signal intensities. No SERS activity is detected on the as-deposited Ag film. All spectra exhibit characteristic vibrational bands attributable to ring breathing modes, C–H bending, and COO^-^ stretching. A detailed peak assignment is provided in Table 1. A representative SERS intensity map of the 10 at% Ag pad is presented in Fig. S4. The map reveals a generally uniform signal distribution across the analysed area, with only minor regions exhibiting reduced intensity.

**Table 1:** Raman peaks observed in SERS spectra of 4-MBA. The peaks were assigned according to previous literature [30, 44, 45]

Peak position [rel. cm^-1^]	Assignment
1078	in-plane C–C ring vibration
1180	C–H bending
1370	COO^-^ stretching
1587	C=C ring vibration

In Fig. 2d, the intensity of the C=C ring vibration is plotted as a function of alloy composition (lower x-axis) for np Ag samples and as a function of surface roughness (upper x-axis) for roughened Ag films. The trend observed in the spectral data is corroborated by this analysis: both EHD-RP and PVD samples show increased SERS intensities with decreasing Ag content, while roughened Ag films display enhanced signal strength with increasing surface roughness. However, it is noteworthy that the SERS signal intensities of roughened Ag films remain approximately an order of magnitude lower than those obtained from np Ag structures. The corresponding EFs, calculated for the C=C ring vibration using 1, are shown in Fig. 2e. As described in section 2, the EF calculation is based on the SERS signal intensity and the effective surface area illuminated by the laser, with the relevant values compiled in Table 2. Pure 4-MBA powder served as the reference for the Raman measurements; the corresponding spectrum is shown in Fig. S5. The calculated EFs reveal consistent trends with both alloy composition and surface roughness. For EHD-RP-derived np Ag, the EF increases from approximately 0.12$\times$10^4^ for the 40 at% Ag sample to 2.64$\times$10^4^ for the 10 at% Ag sample, with a slight reduction observed at 20 at% Ag. Similarly, for PVD-derived np Ag, the EF rises from 0.89$\times$10^4^ at 30 at% Ag to 1.77$\times$10^4^ at 10 at% Ag. In the case of roughened Ag, the EF increases from 0.11$\times$10^4^ after 10 s of roughening to 0.65$\times$10^4^ after 37 s.

**Table 2:** SERS intensities of the symmetric C=C vibrations, surface area in the laser volume and calculated EFs for all samples

Sample	C=C SERS intensity [a.u.]	Surface Area in Laser Volume [$\mu$m^2^]	EF
40at% Ag EHD-RP	0.16±0.11$\times$10^3^	5.75	0.12±0.08$\times$10^4^
30at% Ag EHD-RP	1.75±0.30$\times$10^3^	5.06	1.50±0.25$\times$10^4^
20at% Ag EHD-RP	1.91±0.58$\times$10^3^	7.36	1.12±0.34$\times$10^4^
10at% Ag EHD-RP	3.46±0.29$\times$10^3^	5.68	2.64±0.22$\times$10^4^
30at% Ag PVD	1.87±0.83$\times$10^3^	9.06	0.89±0.40$\times$10^4^
20at% Ag PVD	1.51±0.62$\times$10^3^	6.33	1.03±0.42$\times$10^4^
10at% Ag PVD	2.77±0.35$\times$10^3^	6.78	1.77±0.22$\times$10^4^
10 s roughened Ag	22±9	0.53	0.11±0.04$\times$10^4^
30 s roughened Ag	72±34	0.54	0.35±0.17$\times$10^4^
37 s roughened Ag	140±61	0.56	0.65±0.28$\times$10^4^

An increase in SERS signal intensity for np Ag with decreasing Ag content in CuAg alloys has been previously reported and attributed to differences in the resulting np network structure, particularly the formation of smaller pores that facilitate a higher density of electromagnetic hot-spots [46]. Similar composition-dependent enhancements have also been observed in other alloy systems. For instance, in a CuZrAlAg alloy, the composition containing 10 at% Ag exhibited the highest SERS enhancement, which was attributed to the specific np network morphology at this composition that favoured the formation of hot-spots [47]. A similar trend has been reported for roughened Ag films, where an increase in surface roughness results in enhanced SERS intensities [48]. In the present study, np Ag samples exhibit EFs on the order of 10^4^, with EHD-RP samples generally yielding slightly higher values than those produced via PVD. An exception is observed for the EHD-RP sample with 20 at% Ag, which displays a lower EF compared to other compositions. As shown in Table 2, this sample possesses a comparatively higher surface area within the laser volume than other EHD-RP-derived np Ag samples. The calculation of this surface area, as described in section 2, is based on three primary assumptions. First, it is assumed that the FIB tomography volume is representative of the overall structure. Literature suggests that the minimum size of a representative volume should be at least $(feature size\times 15)^3$ to ensure statistical relevance for structural analysis [49]. In this study, the relevant feature size is defined as pores smaller than 25 nm, as elaborated in the subsequent section. This corresponds to a minimum representative volume of 0.05 $\mu$m^3^. The FIB reconstructions used in this work have a volume of approximately 1.7 $\mu$m^3^, thereby satisfying this criterion. Second, the laser interaction volume was approximated as a sphere with a diameter of 811 nm, based on confocal microscopy parameters detailed in section 2. This simplification does not perfectly capture the actual shape of the focal volume. It has been shown in literature that the laser volume is very localised, but deviates from perfect spherical symmetry [50]. The associated error is expected to be systematic across all samples. Consequently, it does not compromise the validity of the relative comparison between samples. Third, it is assumed that the entire laser volume resides within the bulk of the np Ag sample. To ensure this, focus alignment was performed at the sample surface followed by upward stage adjustment to embed the focal volume within the material. Given the elevated surface area measured for the 20 at% Ag sample the value for $N_{SERS}$ will also increase resulting in a lower calculated EF. To determine whether this elevated surface area is an intrinsic characteristic of EHD-RP samples at this composition, further FIB tomography measurements for this sample would be required. Reported EFs for np Ag in the literature typically range from 10^5^ to 10^6^ [18, 31, 47, 51]. In more specialised cases, such as rhodamine 6 G on np Ag, EFs as high as 10^8^ have been reported, attributed to enhanced charge-transfer interactions between the molecule and the Ag surface [52]. The EFs obtained in this work are approximately one to two orders of magnitude lower than those reported previously. This discrepancy may stem from differences in the surface coverage of the probe molecule 4-MBA. Although a surface coverage of approximately 0.5 nmol cm^-2^ for 4-MBA is often cited [30, 31], these estimates are taken from calculations for thiophenol [53], and may not fully account for the additional interactions introduced by the carboxyl functional group. Specifically, the pH-dependent chemisorption of 4-MBA on Ag surfaces has been shown to involve both S and COO^-^ binding modes at neutral and alkaline pH [54, 55]. The presence of the COO^-^ vibrational peak in all spectra (Fig. 2) suggests that 4-MBA molecules adopt a more tilted orientation on the surface. Such a tilting would reduce molecular packing density and thereby decrease surface coverage, leading to an overestimation of $N_{SERS}$. The true value of $N_{SERS}$ is most likely lower, increasing the calculated EF, potentially reconciling the differences between the measured and literature values. When comparing the obtained EFs to those reported for other SERS substrates, it becomes evident that our values lie on the lower end of the typical range. For nanoparticle-based substrates, reported EFs span approximately from 10^4^ to 10^11^, with the majority of studies reporting values around 10^8^ [56–60]. Lithographically fabricated nanostructures generally exhibit a narrower distribution, typically ranging from 10^4^ to 10^8^ [61–64]. The observed increase in EF with surface roughness for Ag films is consistent with previously reported trends, which place such EFs in the range of 10^3^ to 10^4^ [65]. The values obtained in our study for roughened Ag align well with these reports. The absence of any measurable SERS signal from the as-deposited Ag film is in agreement with the lack of nanoscale surface features capable of producing LSPRs. In this context, roughened Ag serves as a practical benchmark for evaluating np Ag substrates. Both EHD-RP and PVD-derived np Ag samples outperform roughened Ag by a factor of approximately four in terms of SERS enhancement, underscoring their potential utility as high-performance SERS substrates. These results reinforce the significance of nanoscale morphology and surface structuring in determining SERS activity and shows that np Ag shows similar promise independent on the fabrication method.

### Structural factors influencing SERS enhancement

**Fig. 3:**
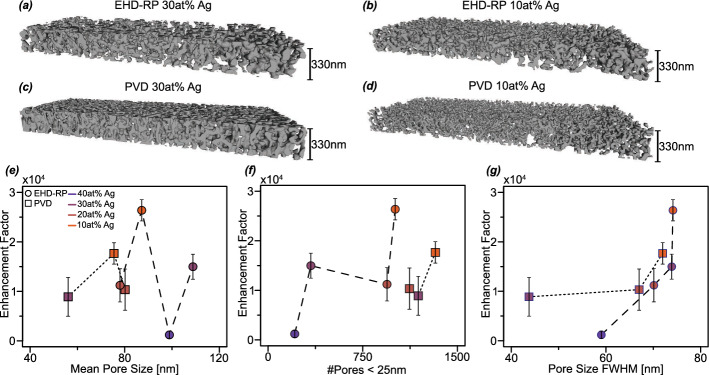
Structural analysis of np Ag networks and its influence on EFs. **a, b** 3D reconstruction from FIB tomographies of EHD-RP np Ag samples with a starting Ag content of 30 and 10 at% respectively.** c, d** 3D reconstruction from FIB tomographies of PVD np Ag samples with a starting Ag content of 30 and 10 at% respectively. **e–g** Calculated EFs of np Ag networks in dependence on the mean pore size, number of pores smaller than 25 nm and pore size distribution FWHM, respectively. Round data points are EHD-RP samples and rectangular data points are PVD samples. The composition is also illustrated in the colour of data points where purple corresponds to a higher Ag content and orange to a lower Ag content

To investigate structural differences among the np Ag samples, FIB tomography was used. Figure 3a–d presents the three-dimensional reconstructions of the np Ag networks obtained from EHD-RP and PVD samples with initial Ag contents of 30 and 10 at%. In both sample types, a reduction in Ag content results in a discernible decrease in ligament size, accompanied by an increase in the apparent structural disorder of the network, an effect particularly pronounced in the PVD-derived samples. Reconstructions for additional compositions are provided in Fig. S6. These FIB tomographies further allow for quantitative analysis, which can be correlated with EFs of np Ag samples. When looking at the measured mean pore size, as shown in Fig. 3e, it is apparent that it does not correlate with the EFs. However, when only pores under 25 nm are considered, a trend can be observed. As illustrated in Fig. 3f, an increase in the number of small pores correlates with an increase in EF for both EHD-RP and PVD-derived np Ag. However, the EHD-RP sample with 20 at% Ag constitutes a notable deviation from this trend, exhibiting a slightly lower EF than the 30 at% Ag sample despite possessing a greater number of small pores. To account for the influence of the degree of disorder the full width at half maximum (FWHM) of the pore size distribution can be taken as a qualitative proxy [66–68]. For both EHD-RP and PVD samples an increase in FWHM is associated with higher EFs, as depicted in Fig. 3g.

Comparative analysis of the tomographic three-dimensional reconstructions for EHD-RP and PVD samples with 30 at% Ag reveals that the PVD-derived network exhibits a higher density and an apparently greater degree of structural ordering. This observation is consistent with the trends identified from the SEM micrographs presented in Fig. 1. A comparison between the 30 and 10 at% Ag samples further highlights a pronounced reduction in ligament size and an increase of disorder with decreasing Ag content. The reduction in ligament dimensions with decreasing noble metal concentration has been previously reported and attributed to accelerated dealloying kinetics in alloys with lower noble metal content [37, 69, 70]. This behaviour is corroborated by the dealloying current measurements for PVD samples shown in Fig. S3, where the 30 at% Ag sample required approximately 3000 s for complete dealloying, compared to only 650 s for the 10 at% Ag sample. Additionally to the influence on ligament size, prior studies have also shown that the alloy composition can influence the overall network morphology and connectivity[71–73]. However, to the best of our knowledge, the specific impact of composition on the degree of disorder within the network has not been previously explored. The results presented here suggest that lower noble metal content may lead to increased structural disorder in the np network. Nonetheless, confirming this trend with high confidence would require further investigation, which lies beyond the scope of this study. To understand the influence of np Ag network morphology on EFs, several structural parameters were evaluated. Firstly, the mean pore size was considered. Although prior studies have reported a correlation between smaller mean pore sizes and enhanced SERS activity for np metals (see section 1), no such trend is evident from the data presented here, as shown in Fig. 3e. However, when focusing exclusively on pores smaller than 25 nm, a clear dependence emerges: as shown in Fig. 3f, the EF increases with the number of sub-25 nm pores for both EHD-RP and PVD samples. The only deviation from this trend is the 20 at% Ag EHD-RP sample, which exhibits a lower EF despite a relatively high number of small pores. The strong influence of small pores in SERS enhancement is consistent with previous findings from nanoparticle systems, where significant signal amplification was observed only when inter-particle gap sizes were reduced below approximately 30 nm [74, 75]. Finally, the degree of structural disorder within the np Ag networks was investigated. While direct experimental quantification of disorder remains challenging, the FWHM of the pore size distribution can serve as a useful proxy, with larger FWHM values indicating a higher degree of disorder [66–68]. As shown in Fig. 3g, an increase in FWHM correlates with an increase in EF for both EHD-RP and PVD samples. Notably, the EHD-RP sample with 20 at% Ag adheres to this trend, in contrast to its behaviour with respect to the number of small pores. Prior literature has also reported a positive correlation between structural disorder and SERS performance, as discussed in section 1. These findings collectively underscore the multifaceted nature of the relationship between np Ag morphology and SERS enhancement, highlighting the critical role of nanoscale features in dictating optical performance.

### Stability of np Ag networks under laser irradiation

**Fig. 4:**
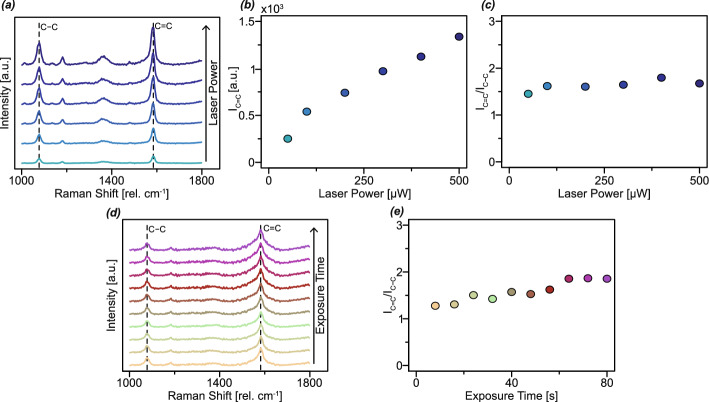
Stability of SERS measurements with increasing laser power and laser exposure time. **a** SERS spectra of 4-MBA acquired with varying laser power on np Ag from a 10 at% Ag EHD-RP sample. **b** SERS intensity of the C=C peak as a function of the laser power. **c** SERS intensity ratio of the C–C and C=C peaks at varying laser powers. **d** SERS spectra of 4-MBA acquired with varying laser exposure time on np Ag from a 10 at% Ag EHD-RP sample. (**e**) SERS intensity ratio of the C–C and C=C peaks at varying laser exposure time

To evaluate the stability of np Ag SERS substrates at varying laser power and exposure time, series of SERS measurements were conducted on np Ag derived from 10 at% Ag EHD-RP samples. These samples were selected based on their previously determined high EFs. The influence of laser power on SERS spectra is presented in Fig. 4a–c. Measurements were carried out using laser powers ranging from 50 to 500 $\mu$W. An increase in laser power results in a corresponding increase in signal intensity. When plotting the signal intensity as a function of the laser power it can be seen that this increase follows a linear trend. The spectral features and relative peak intensities are not affected by the laser power, as illustrated by the stable ratio of the C–C to C=C peak intensities. Changes in the intensity ratio of these peaks can give an insight into the the enhancement mechanisms [76]. To assess the stability over time of the substrates under laser irradiation, additional measurements were performed at a constant laser power of 200 $\mu$W with increasing exposure times, as shown in Fig. 4d, e. Even after 80 s of continuous exposure, no degradation or shift in spectral peaks was observed, demonstrating the high photostability of the np Ag network. A slight increase in the C–C/C=C peak intensity ratio was noted with prolonged exposure, with a maximum increase of approximately 25% after 80 s.

Np Ag substrates fabricated via EHD-RP exhibit high stability under laser irradiation. The linear increase in signal intensity with an increase in laser power highlights the structural stability of the fabricated np Ag networks. Even at elevated laser powers of up to 500 $\mu$W (approximately 240 $\mu$W $\mu$m^-2^), no discernible changes are observed in the SERS spectra, confirming the structural robustness of the np network even further. Likewise, extended laser exposure at 200 $\mu$W does not lead to any spectral degradation, with peak positions and intensities remaining consistent throughout the measurement duration. However, the slight shift in the C–C/C=C peak intensity ratio indicates some changes in the system. This change in peak intensity ratio can be attributed to several factors. First, the ratio between a non-totally symmetric vibrational mode (in this case, the C–C vibration) and a totally symmetric mode (the C=C vibration) has been linked to the relative contributions of EM nd CT enhancement mechanisms in SERS [76]. Second, the relative intensities of SERS peaks can also be influenced by the orientation of the adsorbed probe molecules with respect to the local electromagnetic field [77]. Given the predominantly random orientation of ligaments within the np network, it can be assumed that the orientation of adsorbed probe molecules is likewise largely isotropic. A more comprehensive evaluation of molecular orientation effects could be achieved through polarisation-dependent SERS measurements. However, such an investigation falls outside the scope of the present study. Both the above described effects may be sensitive to local temperature variations at the substrate surface, particularly since laser-induced heating of nanoporous metals has been previously reported [78, 79]. Despite these minor spectral variations, the absence of any peak shifts or significant changes in intensity confirms that no substantial structural alterations occur within the np Ag network under the investigated optical conditions. These findings underscore the excellent structural stability of EHD-RP-derived np Ag, reinforcing its suitability as a reliable SERS substrate for practical applications.

### Catalytic activity

**Fig. 5:**
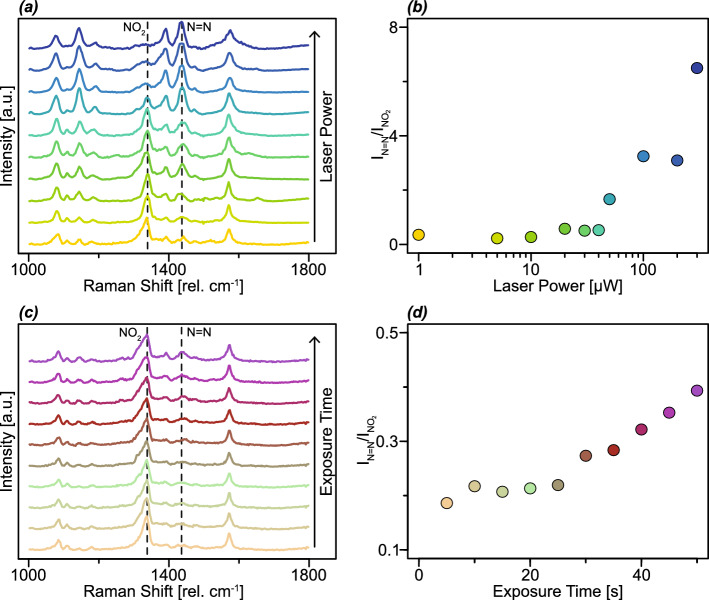
Plasmonically catalysed 4-NBT dimerisation reaction. **a** SERS spectra of 4-NBT acquired with varying laser power on np Ag from a 10 at% Ag EHD-RP sample. The spectra were normalised to their maximum intensity to enable better comparability. **b** SERS intensity ratio of the N=N and $\textrm{NO}_2$ peaks with varying laser power. **c** SERS spectra of 4-NBT acquired with varying laser exposure time on np Ag from a 10 at% Ag EHD-RP sample. **d** SERS intensity ratio of the N=N and $\textrm{NO}_2$ peaks with varying laser exposure time

To evaluate the catalytic performance of EHD-RP-derived np Ag in plasmonically driven chemical reactions, SERS measurements were carried out using 4-NBT as a probe molecule. It is well established that 4-NBT undergoes a plasmon-assisted dimerisation reaction to form *p,p’*-dimercaptoazobenzene (DMAB) [80–82]. The dependence of this transformation on laser power is illustrated in Fig. 5a and b. With increasing laser power, a clear rise in the intensity of characteristic DMAB peaks is observed, as identified in Tab. 3. This trend is further quantified by evaluating the N=N/$\textrm{NO}_2$ peak intensity ratio. The N=N peak at 1437 cm^-1^ is specific to DMAB, while the $\textrm{NO}_2$ peak at 1338 cm^-1^ is specific to 4-NBT. Therefore, this intensity ratio can be used as an indicator for the propagation of the dimerisation reaction. The ratio remains relatively constant at low laser powers but begins to increase sharply at powers above 50 $\mu$W, indicating enhanced conversion of 4-NBT to DMAB. Notably, even at low laser powers such as 1 $\mu$W, the reaction proceeds over time, as shown in Fig. 5c and d. After only 50 s of laser exposure, the N=N/$\textrm{NO}_2$ intensity ratio increases by approximately 110%.

**Table 3:** Raman peaks observed in SERS spectra of 4-NBT and DMAB. The peaks were assigned according to previous literature[81–83]

Peak Position [rel. cm^-1^]	Assignment	Probe Molecule
1080	In-plane C-C ring vibration	4-NBT, DMAB
1110	C–H bending	4-NBT
1144	C–N stretching	DMAB
1188	C–H bending	DMAB
1338	$\textrm{NO}_2$ vibration	4-NBT
1392	N=N stretching	DMAB
1437	N=N stretching	DMAB
1574	C=C ring vibration	4-NBT, DMAB

The enhanced progression of the 4-NBT dimerisation reaction with increasing laser power, as evidenced by the rising N=N/$\textrm{NO}_2$ peak intensity ratio in Fig. 5b, is consistent with previous findings in literature [81, 82]. It has further been demonstrated that the excitation wavelength significantly influences the reaction rate, with lower wavelengths leading to more rapid conversion [82, 83]. For this reason, comparison is made specifically to results obtained at 532 nm, the excitation wavelength also used in this study. In previous work conducted at 532 nm, it was reported that complete conversion to DMAB occurred after 300 s of irradiation at a laser power of 500 $\mu$W, with the authors noting that this power was already considered low for initiating the reaction [82]. The data we present in Fig. 5b shows that EHD-RP derived np Ag can catalyse the reaction efficiently at laser powers as low as 50 $\mu$W. With an increased laser exposure time progression of the reaction can even be observed for laser powers as low as 1 $\mu$W, as illustrated in Fig. 5d. This highlights the exceptional catalytic performance of EHD-RP-derived np Ag and underscores its strong potential for applications in plasmon-driven chemical reaction under mild optical conditions.

## Conclusions

In summary, this work demonstrates that np Ag fabricated via EHD-RP performs comparably to conventionally deposited np Ag from PVD in terms of SERS performance, and outperforms roughened Ag films. For both fabrication methods, the initial alloy composition was found to strongly influence the achievable EFs, with lower Ag content resulting in higher signal enhancement. FIB tomography revealed that specific structural parameters govern the SERS activity of np Ag. Notably, a higher density of small pores – rather than a reduction in mean pore size alone – was correlated with increased EFs. Additionally, a greater degree of structural disorder, as indicated by the FWHM of the pore size distribution, also contributed to enhanced signal intensities. The EHD-RP-derived np Ag samples exhibiting the highest SERS activity further demonstrated excellent stability under increased laser powers and prolonged laser exposure times. Moreover, these structures were capable of catalysing the plasmon-driven dimerisation of 4-NBT to DMAB at laser powers as low as 1 $\mu$W, highlighting their catalytic potential. These findings collectively underscore the suitability of EHD-RP-derived np Ag as both robust SERS substrates and efficient plasmonic catalysts. Owing to the method’s high spatial resolution and compositional control, EHD-RP presents a promising avenue for integration into micro- and nanofabrication applications, particularly in microfluidic systems, where localised material deposition remains a significant challenge [84]. Moreover, the three-dimensional design flexibility of EHD-RP allows the creation of architectures exhibiting plasmonic resonances originating from their geometry [85, 86], which may be combined with the intrinsic resonances of the np network to enhance optical performance.

## Additional file

Supplementary file 1 (pdf 1478 KB)

## Data Availability

The raw/processed data required to reproduce these findings can be requested from the corresponding author.
