# Supplementary material for: Electrohydrodynamic redox printing vs. physical vapour deposition: a comparative study of nanoporous Ag morphology and SERS performance
Source: Discov Nano. 2025 Dec 9;20(1):228. doi: 10.1186/s11671-025-04409-1 (PMC12690026; doi:10.1186/s11671-025-04409-1)
Supplement: Supplementary file 1 — (pdf 1478 KB) [file 11671_2025_4409_MOESM1_ESM.pdf]

Supplementary Information

Title

Nikolaus Porenta, Loredana Piazza and Ralph Spolenak

1 Materials and Methods

In Fig. S1 a CV scan of a mixed 8 mM $CuSO_4$ and 1 mM Ag_2SO_4 solution in 10 vol% H_3PO_4 is shown. Peaks can be observed at approximately −0.31, −0.18, 0.08 and 0.16 V. The correspond to the reduction of Cu, oxidation of Cu, reduction of Ag and oxidation of Ag, respectively.

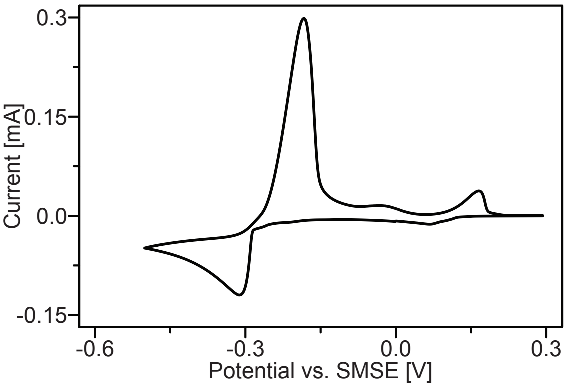

Fig. S1 CV scan of a mixed 8 mM $CuSO_4$ and 1 mM Ag_2SO_4 solution in 10 vol% H_3PO_4.

2 Results and Discussion

Tab. S1 Composition of the used starting alloys, measured using SEM EDX.

Sample	Ag content [at%]	Cu content [at%]
40at% Ag EHD-RP	43.84±1.90	56.16±4.46
30at% Ag EHD-RP	28.31±1.39	71.69±5.26
20at% Ag EHD-RP	18.46±1.87	81.54±5.83
10at% Ag EHD-RP	10.98±0.82	89.02±5.65
30at% Ag PVD	30.39±1.29	69.61± 5.08
20at% Ag PVD	20.05 ± 0.92	79.95 ± 5.45
10at% Ag PVD	9.71±0.60	90.29 ± 5.60

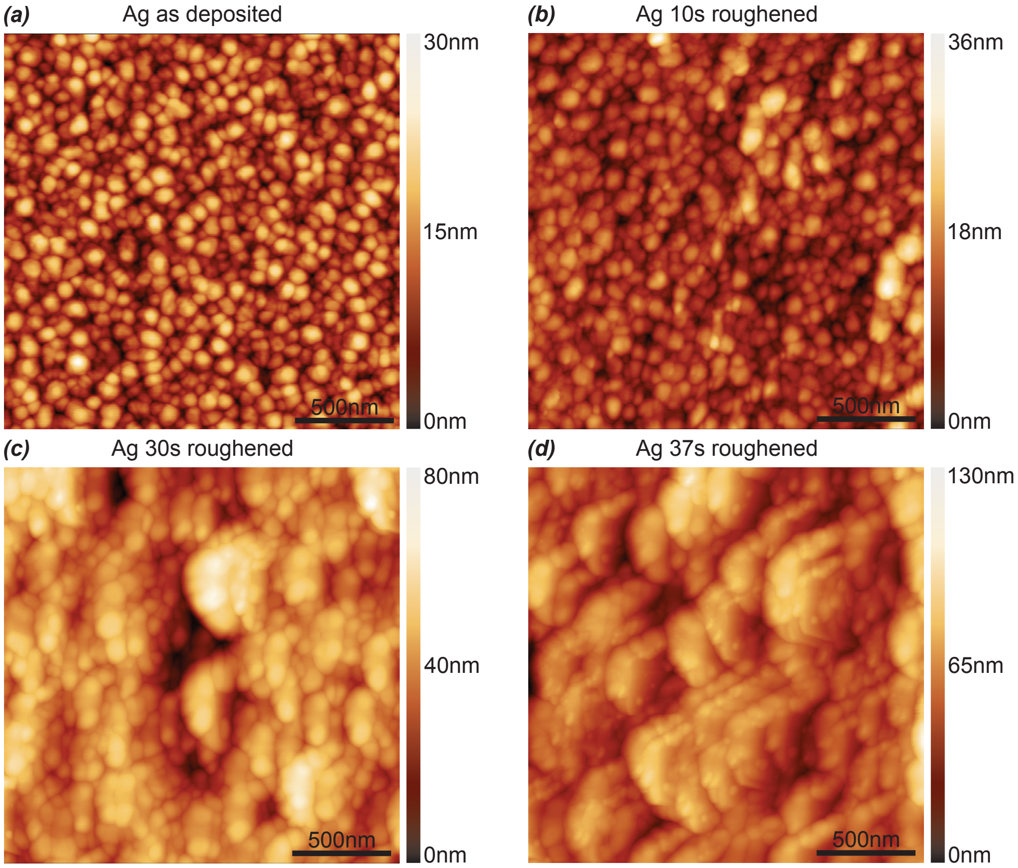

Fig. S2 AFM micrographs of Ag films (***a***) as-deposited and (***b***-***d***) roughened for 10, 30 and 37 s respectively.

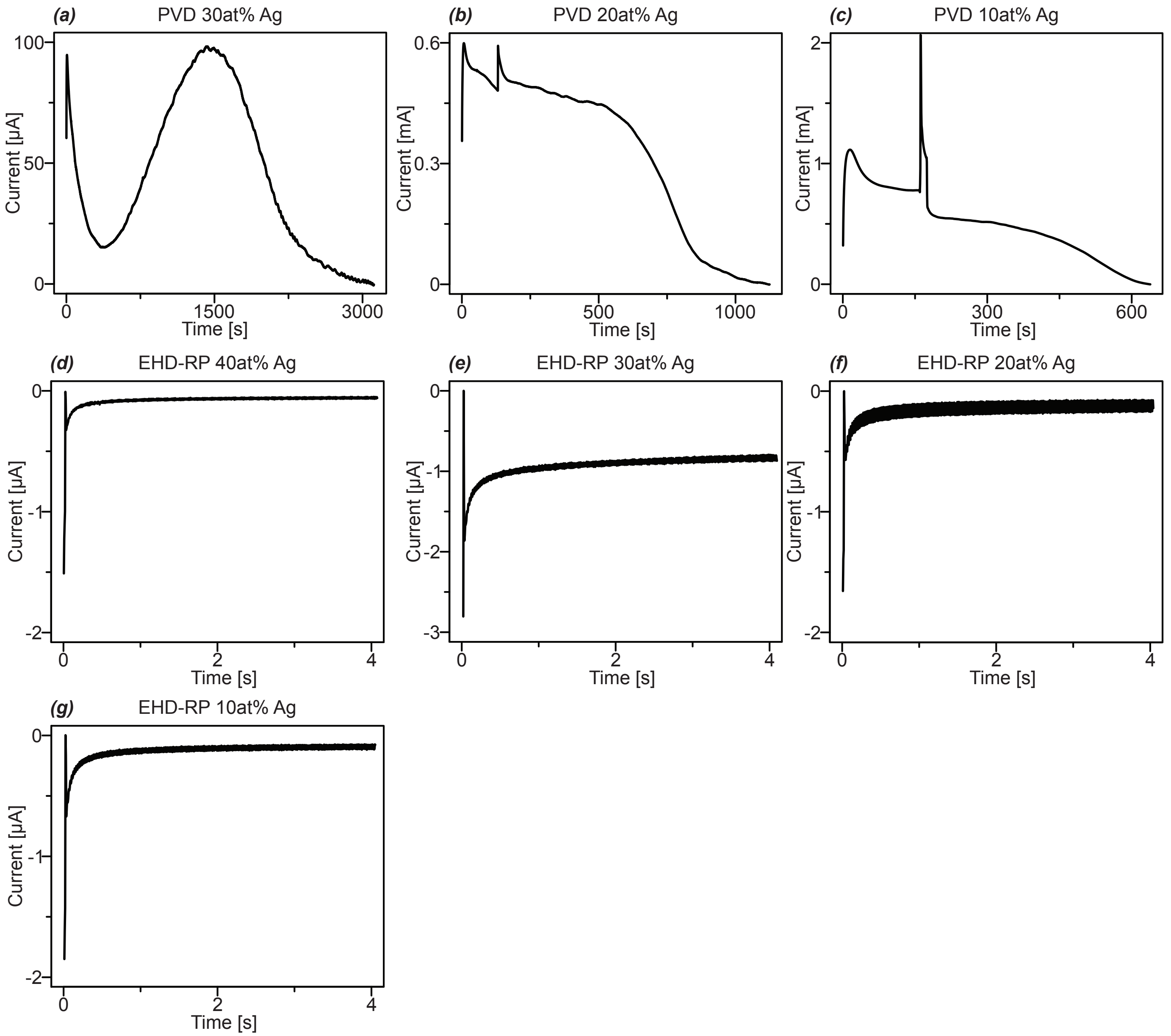

Fig. S3 Dealloying current measurements for (***a***-***c***) PVD samples with 30, 20 and 10 at% Ag and (***d***-***g***) EHD-RP samples with 40, 30, 20 and 10 at% Ag. The sharp peaks visible in (***b***-***c***) were caused by vibrations on the measurement table.

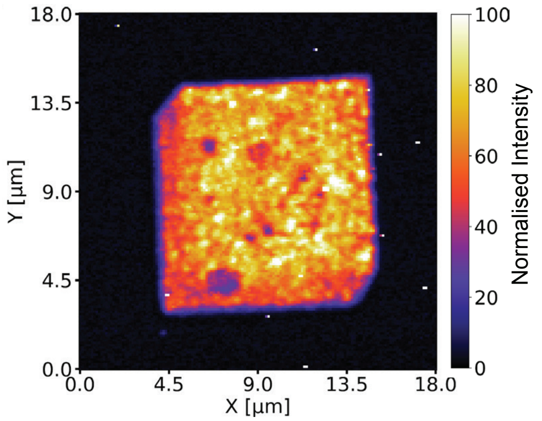

Fig. S4 SERS map of the symmetric C=C vibrational mode on a 10 at% Ag EHD-RP sample.

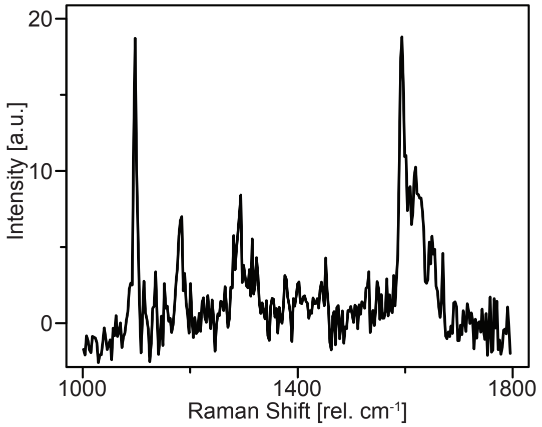

Fig. S5 Raman spectrum of pure 4-MBA powder.

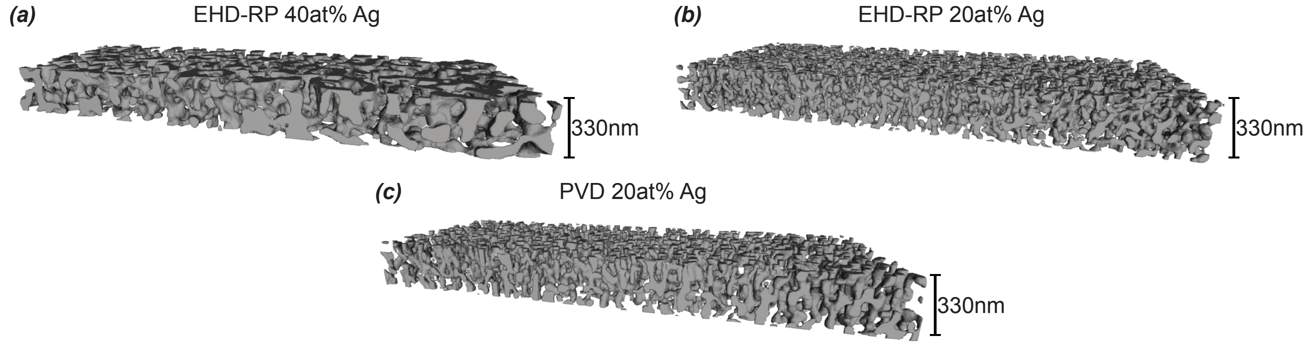

Fig. S6 (***a-b***) 3D reconstruction from FIB tomographies of EHD-RP np Ag samples with starting Ag compositions of 40 and 20 at% respectively. (***c***) 3D reconstruction from FIB tomographies of PVD np Ag with a starting Ag compositions 20 at%.
